# Glucocorticoid metabolites in an *ex situ* nocturnal bird, the tropical screech owl *Megascops choliba*: effects of sex, activity period and inter-individual variation

**DOI:** 10.1093/conphys/coad016

**Published:** 2023-04-22

**Authors:** Heriberto Barbosa-Moyano, Gisela Sobral, Claudio Alvarenga de Oliveira

**Affiliations:** Departamento de Reprodução Animal, Faculdade de Medicina Veterinária e Zootecnia, Universidade de São Paulo, Av. Prof. Dr. Orlando Marques de Paiva, 87, CEP: 05508270, São Paulo (SP), Brazil; Instituto de Biodiversidade e Sustentabilidade NUPEM/UFRJ, Av. São José do Barreto, 764–São José do Barreto, Macaé – Rio de Janeiro (RJ), 27965-045, Brazil; Departamento de Reprodução Animal, Faculdade de Medicina Veterinária e Zootecnia, Universidade de São Paulo, Av. Prof. Dr. Orlando Marques de Paiva, 87, CEP: 05508270, São Paulo (SP), Brazil

**Keywords:** Strigiformes, immunoassay validation, daily rhythm, behavioural endocrinology

## Abstract

Glucocorticoids mediate physiological processes to obtain energy, presenting daily variation in basal levels that may be related to behavioural activity pattern. Identification of plasticity in the secretion of these hormones is essential to understand their effects on physiology and behaviour of wild birds and, therefore, their success in their natural or artificial environment. Serial endocrine evaluations are facilitated by implementing non-invasive methodologies that minimize possible effects of manipulation on the animal’s physiological variables. However, non-invasive endocrine-behavioural studies in nocturnal birds, such as owls, are immature. The present work aimed to validate an enzyme immunoassay (EIA) to quantify glucocorticoid metabolites (MGC) in *Megascops choliba* as well as to evaluate differences in their production at the individual, sexual or daily level. We recorded the behaviour of nine owls during three continuous days to establish activity budget under captive conditions and aiming to correlate with daily MGC variation. The EIA proved to be effective in analytical assays and in pharmacological testing with synthetic ACTH, validating this immunoassay for the species. Additionally, individual differences in MGC production were confirmed in relation to the time of day, especially at 1700 and 2100, but not in relation to sex. During night hours, the owls showed greater behavioural activity, positively related to MGC values. Higher MGC concentrations were significantly related to greater expressions of active behaviours, such as maintenance, while lower MGC concentrations were recorded during moments of higher alertness and resting. The results presented show daily MGC variation to be inversed in this nocturnal species. Our findings can aid future theoretical studies of daily rhythm and evaluations of challenging and/or disturbing situations that result in changes in behaviour or hormonal cascades of these changes in *ex situ* populations of owls.

## Teaser

This study analytically and biologically validated an enzyme immunoassay for quantification of glucocorticoid metabolites (MGC) in *Megascops choliba*. Differences in MGC levels were recorded between individuals in relation to time of day, but not in relation to sex. Additionally, higher frequency of active behaviours was related to higher levels of MGC.

## Introduction

*Ex situ* populations of wild species stand out as a unique opportunity to shed light on the still insufficiently understood hormone-behaviour relationship in nocturnal animals. Such knowledge gap derives from inherent difficulties to find and recover samples in the wild (Trolliet *et al.,* 2014; Alonso *et al.,* 2021), as well as the labor-intensive or unfeasible repeated capture of the same individuals and low-density occurrence of many species (Schemnitz *et al.,* 2009; Newton *et al.,* 2016).

Wild animals under human care are often exposed to *ex situ* conservation practices, such as management, shelter, care and breeding carried out by entities like zoos, aquariums, as well as reproduction and rehabilitation centers (Maxted, 2013; McGowan *et al.,* 2017; Mestanza-Ramón *et al.,* 2020). Exposure to such handling procedures may act as psychological stressor (Fischer and Romero, 2018), potentially resulting in behavioural alterations and consequent changes in glucocorticoid levels (Cockrem, 2013, 2022; McCormick and Romero, 2017), impairing reproduction and welfare (Narayan, 2020).

Cortisol and corticosterone are glucocorticoid hormones involved in physiological process regulation, such as energy acquisition, storage and mobilization (McEwen and Wingfield, 2003; Busch and Hayward, 2009). However, their production is altered after individuals are exposed to disturbing situations, such as agonistic interactions and predator presence (Romero and Beattie, 2022), the so-called fight-or-flight response (Cannon, 1939). Hence, glucocorticoids (GCs) exert a physiological and behavioural mediating effect on allostatic processes both during predictable activities and unpredictable and/or challenging situations (Mastorakos *et al.,* 2005; Landys *et al.,* 2006; Therrien *et al.,* 2008) that go beyond those associated with a response to stressors (MacDougall-Shackleton *et al.,* 2019).

Glucocorticoids synthesis by the adrenal glands is pulsatile and fine-tuned by environmental factors, such as daily photodynamic variation, corresponding to the circadian rhythm (Boissin and Assenmacher, 1970; Albrecht, 2013; Nicolaides *et al.,* 2015). In diurnal species, the peak occurs towards the end of the dark period, whilst in nocturnal species, peak is towards the end of the light period (Touma and Palme, 2005). Therefore, regular variation in GC levels is still generally associated with energy-expenditure activities (e.g. locomotion and reproduction) or energy acquisition (e.g. foraging and resting) (Romero *et al.,* 1997; Busch and Hayward, 2009; Casagrande *et al.,* 2018).

There might be other biologically relevant variations in GCs, such as those provided by specific life-history stages and even individual variation. For instance, basal GC levels are higher during establishment of breeding territories (Romero *et al.,* 1997) and during natal dispersal (Holekamp and Sherman, 1989; Belthoff and Dufty, 1998). Additionally, GC production, synthesis and metabolism may differ between sexes (Goymann, 2005; Casagrande *et al.,* 2018). Such differences may correlate with distinct reproductive investment and/or parental care provided by each sex. In species with bi-parental care, similar GC levels are expected for both sexes (Harris *et al.,* 2013), although there may be differences in daily GC rhythms for each sex (Koch *et al.,* 2002). Finally, although GCs is generally considered as a predictable response at population level, recent studies are discovering important inter-individual variation (Madliger and Love, 2013; Ouyang *et al.,* 2013; Love *et al.,* 2014). Many of these studies are detecting that personality is an important factor in the association between glucocorticoids and behavioural response (Raulo and Dantzer, 2018).

Assessment of glucocorticoids concentration and/or their metabolites (MGC) variation, combined with behavioural analysis, provides tools to understand the biology, ethology and ecology of the species, which are defining aspects of animal welfare programs (Palme, 2019). Some papers have found a relationship between high MGC concentration and self-injuring behaviours in wild mammals and birds kept in captivity (Wielebnowski *et al.,* 2002; Pizzutto *et al.,* 2015; Costa *et al.,* 2016). Conditions for this include specific management activities, small-sized enclosures or lack of enrichment (Wielebnowski *et al.,* 2002; De Almeida *et al.,* 2018). Despite the usefulness of combining behavioural assessment with non-invasive endocrine monitoring, few studies have integrated these variables in wild birds kept captive (Fairhurst *et al.,* 2011; Haase *et al.,* 2021; Woods *et al.,* 2022). Such gap is particularly pronounced for owls; although some studies associated MGC with stressful events (Wasser *et al.,* 1997; Tempel and Gutiérrez, 2004; Wasser and Hunt, 2005; Béziers *et al.,* 2020), they did not link their results with behavioural evaluation.

About 250 species of owls are distributed worldwide (König *et al*., 2008), and many of them are in some degree of conservation threat. Like many other taxa, Neotropical owls are understudied compared to temperate climate owls (Enríquez *et al.,* 2017). A proper knowledge of their behavioural endocrinology is lacking. The aim of the present paper is to provide a behavioural endocrinology assessment of a nocturnal bird and understand the natural sources of GC variation, namely daily activity, sex and individual differences. Our animal model is the tropical screech owl (*Megascops choliba*), a widely distributed species in the American continent of Least Concern conservation status (BirdLife, 2021). Hence, research in this species addressing behavioural and endocrine issues will aid the development of conservation methodologies for other species of nocturnal raptors. The present work describes the activity budget of a tropical screech owl under captive conditions. We tested the hypothesis that higher behavioural activity is associated with the variation of glucocorticoid metabolites. Specifically, we aim to (i) validate an EIA for detection and quantification of MGC; (ii) assess the possible difference in MGC production at the individual, sexual or circadian level; (iii) estimate activities budget; and finally, (iv) evaluate possible relationship between MGC concentrations and activity budget.

## Materials and Methods

### Ethical note

The procedures proposed in this study were approved by the ethics committee regarding the Use of Animals—CEUA of FMVZ-USP process no. 6896290419 and had authorization from SISBIO/IBAMA (no. 70120-1) and SisGen no. A6EEC44. Additionally, the activities that were developed at the Centro de Manejo e Conservação de Animais Silvestres—CeMaCAS were approved and/or authorized according to process no. 60272019/0000675-1.

### Animals and housing

Animals were housed at Centro de Manejo e Conservação de Animais Silvestres (CeMaCAS), São Paulo, Brazil (23° 24 ′47′′S 46° 47′ 29′′W) and were all at their final phase of rehabilitation (pre-release). Six owls were used in the immunoassay validation tests (Aug/2020) and another nine individuals in the quantification of MGC concentrations, together with determination of activities budget (Nov/2020). Birds had a mean weight of 118.3 ± 9.1 g, and experiments were carried out in adult individuals from non-reproductive and non-molting phases. The reproduction of *Megascops choliba* is seasonal (Guilherme and de Souza, 2017), which is being determined for the region of São Paulo (unpublished data). Animals were thoroughly examined one month prior to testing, including serological laboratory tests and elimination of parasites.

We sent blood samples for sex determination to Unigen Tecnologia do DNA (São Paulo, Brazil). Each owl was kept in a galvanized wire cage (70 × 70 × 70 cm), with two perches and visual, acoustic and olfactory communication possible. Water was provided *ad libitum* and they were fed dead mice and quail chicks daily. All cages containing individuals were kept in a separate room (without contact with any other species), and room conditions were natural (temperature between ~ 14°C and 25°C, natural ventilation, and light). Between 19 and 22 November 2020, sunrise was at 0512 and sunset at 1830.

### Measurement of glucocorticoid metabolites

Fecal samples were collected with the help of metal tweezers to avoid urate collection at 4-hour intervals (0900, 1300, 1700, 2100, 0100 and 0500). These intervals were necessary to obtain the minimum amount of feces (±0.050 g). Samples were transferred to a 2-mL tube (Eppendorf®) and stored in −80°C freezer until analysis. Samples were lyophilized for 18 hours using a freeze-drying machine (Liotop model L108). Steroid extraction method followed Scheun *et al.* (2020), with modifications. The lyophilized fecal material was grounded, weighed (±0.050 g) and transferred to a glass tube containing 1.5 mL of 80% methanol. The mixture was vortexed for 5 minutes at 2200 rpm three times and then centrifuged for 10 minutes at 1500 g. With the aid of a pipette, we transferred 1 mL of the supernatant (steroid extract) to a new test tube, and dried it under air flow at 60°C temperature. After drying, 0.3 mL of buffer solution was added (NaH_2_PO_4_, Na_2_HPO_4_, NaCl, BSA, pH 7.0) and homogenized again in vortex. Finally, each sample was diluted in the same buffer solution (1:16) estimating the MGC concentration in duplicate. MGC measurements employed an enzyme-linked immunosorbent assay with polyclonal antibody against corticosterone (CJM006) diluted 1: 4000 in coating buffer (Na_2_CO_3_, NaHCO_3_, pH 9.6) and a corticosterone horseradish peroxidase ligand (diluted 1:40 000 in EIA buffer) (Coralie Munro, UC Davis, CA). Plate absorbance readings were measured at 405 nm in an ELISA spectrophotometer (ELx 808TM Bio Tek Instruments Inc, USA).

### Analytical and physiological validation of the immunoassay

Analytical validation of the EIA was performed through parallelism, accuracy and precision tests. The parallelism test consisted of the comparison of two curves: graphic representation of the diluted concentrations of the corticosterone standard (Sigma-Aldrich C2505) versus the serial diluted curve obtained with the pooled samples. Accuracy was performed by recovery check: a known amount of corticosterone standard was spiked into the equal aliquots of the pooled sample with the 1:16 dilution factor. The amount of corticosterone recovery was then calculated dividing %B observed (spiked sample minus non-spiked sample) by the %B expected (known corticosterone standard). Finally, precision was estimated by calculating intra- and inter-assay variation coefficients (CV).

In the physiological validation of the EIA, we challenged adult animals with a synthetic ACTH analogue (Synacthen, Sigma Chemical no. A-6303). Two individuals (female #90089 and male #91876, hereafter ACTH group) received high and low doses (0.5 and 0.25 mg/kg, respectively, Sinhorini *et al.,* 2020). A second pair (female #92166 and male #88169, hereafter saline group) were treated with 0.15 mL of saline solution and the third, non-manipulated pair (female #91988 and male #90129, hereafter control group) was the control. The ACTH analogue was administered intramuscularly between 1900 and 1930. Handling took under 40 seconds per animal. Fecal samples were collected between 34 hours prior to administration times: t equals −34, −30, −26, −22, −18, −14, −10, −6, −2) and 34 hours post (t equals 2, 6, 10, 14, 18, 22, 26, 30, 34), with injection occurring at moment t0.

### Ethogram and behavioural data collection

Behavioural data collected comprised nine owls (three females and six males) throughout three consecutive days (20–23 November 2020). Ethogram was created based on Barbosa-Moyano *et al.* (2019) and Ramanujam (2015), with modifications to the tropical screech owls. We listed and described 18 behaviours that were later separated between activity and inactivity patterns. Table 1 shows the complete description of each behaviour and its respective category and patterns. Therefore, behaviour category or behaviour pattern in reference to this table will be used. The owls were monitored by an internal CCTV system, composed of two cameras with 720p HD image resolution (model VHL 1120 B; Intelbras, Brazil), coupled to a DVR Multi HD digital video recorder (model MHDX 1104; Intelbras). The cameras had an infrared light system that did not seem to bother the animals.

**Table 1:** List of behaviours exhibited by tropical screech owl *Megascops choliba* in captivity and its respective category of activity budget and activity/inactivity patterns in which they were grouped

**Pattern/Category**	**Behaviour**	**Description**
**Inactivity**		
Rest	Camouflage	Firm and static position of the individual, where head and body movements are almost null. Birds can have their eyes closed or half-open. Sometimes, the bird retracts one leg between the belly plumage while maintaining balance on the perch. Wings folded, and chest may or may not be prominent (puffy). The feathers of the body and head are not erected and/or raised, giving the appearance of a thin bird and auricular tufts can be extended.
Standing	Standing on the perch or the ground	Standing, with both legs on the substrate (cage floor, perch, other) and feathers not ruffled. Wings are folded and the neck is neither stretched nor shrunk. Slight head movements, eyes open.
**Activity**		
Maintenance	Feather ruffle	Body feathers in the upper back, chest and neck regions are slowly ruffled and settled back down.
	Shake out plumage and wings	Quick movements in semicircles, where the bird shakes all the feathers of the body, then lowers the feathers slower than “feather ruffle” behavior. This movement can occur with the feathers of the crown, occiput, neck and throat, or on tail feathers or rectrices.
	Tidying and cleaning the plumage with the beak	Birds arrange their wing, tail, back and belly feathers with their beak. This behavior has no pattern or order and includes opening and lifting one wing at a time to access the feathers below. Tidying up includes removing loose feathers, smoothing and arranging feathers that have become disordered.
	Scratch the feathers	Leaning forward a little, lowering its head and with one of its legs stretched upwards, scratching with its fingers the regions of the neck, head and upper part of the chest, being able to keep the feathers in these regions disheveled. The head and neck regions are rubbed with the toes, performing movements of the paw from back to front.
	Scrape and clean the beak	Standing, the bird brushes its beak sides against some hard surface, alternating the right and left sides against the substrate, which may be the perch itself.
	Cleaning the feet	The fingers are cleaned with the help of the beak, usually after feeding.
	Take a bath	Diving into the container with water. The maximum liquid capacity of these containers is 300 mL, and the individual can stand without wetting the chest. The owl shakes its wings and tail, throwing the water on its body, being able to flex its legs to wet its belly, in addition to performing quick and semicircle movements of its whole body. Dip the head, introducing the beak into the water and wetting the feathers on the back of the head.
	Stretching the wings and legs	Standing on the perch, the bird raises its body. The wings spread and open when they are perched, being able to flap their wings when they remain perched.
Locomotion	Fly	Displacement from one side of the cage to the other, starting from the perches, the floor, or the sides of the cage, involving, necessarily, wing beat.
	Walk	Walks on top of the perch or the ground. May move laterally in short steps.
	Jump	Displacement between perches or to the substrate (ground), where the wings are not fully extended. The movement is the result of the simultaneous thrust of both legs. May have a light wing beat to maintain balance when posing.

**Table 1:** Continued

**Pattern/Category**	**Behavior**	**Description**
Alert	Alert	Standing with the body erect or lowered and looking forward. The head moves in all directions, being able to rotate almost 180 degrees.
Feeding and excretion	Drink water	Standing on the substrate, the bird leans its body forward and stretches its neck so that the beak reaches the water depth, then raises its neck, stretching upwards and swallowing the water.
	Forage	Prey manipulation with their beak or fingers, eating small parts or swallowing it whole. Eyes are sometimes closed during ingestion. It includes moments not only of swallowing, manipulating and transporting the prey.
	Defecate	Urofecal matter is excreted with a slight lifting of the tail feathers.
	Regurgitate	Expulsion by the beak of undigested parts of the prey such as feathers and hair, in the form of an elongated mass.

Videos were recorded continuously between 1700 and 0700, when the owls were active. Between 0700 and 1700, all the owls were mostly in a resting position. Activity budgets were constructed using the scan sampling method (Altmann, 1974), carried out by one observer (H.B.M.). Behaviours were evaluated every 5 minutes, allowing behaviours to be noted up to 15 seconds, the time needed to differentiate the most immobile behaviours. Therefore, 12 scans were collected per individual hourly. We did not include records that occurred during cage-cleaning, which lasted about 15 minutes every four hours, or when the behaviour could not be identified. Fecal samples were collected at 4-hour intervals (0900, 1300, 1700, 2100, 0100 and 0500) and treated with the methods described above. To match the four-hour interval between fecal samplings, activity budgets were also analysed in 4-hour blocks.

### Analysis

For analytical validation of the EIA, parallelism test was performed between the curves of the corticosterone pattern and the tropical screech owl steroid extract pool by means of t-test, evaluating the possible differences between linear and angular coefficients of the log-transformed data. Accuracy percentage was determined by dividing observed concentration by expected concentration, multiplied by 100.

Concentrations of glucocorticoid metabolites contained in feces were expressed in ng/g and were presented as mean values with their respective standard deviation (±SD). Baseline values were considered as those before ACTH administration (times −34 to −2). Peak MGC value was determined when values surpassed baseline plus 2.5 SD.

We tested variables for normality with Shapiro–Wilk test and since our variables were normally distributed, we used linear mixed models (LMM), including MGC values as the response variable, sex and time as fixed effects, and individual identity as a random factor (MGC ~ sex * time + (1|id)). Models that were statistically different based on ANOVA test from each other were chosen based on the lowest AIC value and, preferably, the simplest. We also compared the effect of random effects with 95% bootstrapped confidence interval and the effect of random factor removal on AIC.

To compare behaviours and MGC concentration, we combined activity budgets into 4-hour blocks for each individual, calculating the number of behaviours observed along the 4-hour interval and dividing it by total number of scans. The maximum number of scans per individual was 48 (12 scan times 4 hours equals 48). Considering excretion delay, MGC values were transferred to the previous 4-hour blocks. The MGC levels were used as dependent and independent variables in the LMMs. Impossibility to set cause and consequence between hormone and behaviour variation makes this approach advisable (Becker *et al.* 2002). In each LMM, behaviour patterns or categories (see Table 1) were employed separately. All analyses were run on R software version 4.0.3 considering a significance level of p < 0.05.

## Results

### Analytical and physiological validation of the immunoassay

Pool curve slope (slope = −31.01) was statistically similar to that of corticosterone standard curve (slope = −29.74, F = 0.12, df = 1, p = 0.724). Therefore, immunogenic similarity between standard antigens and sample antigens showed parallelism, validating the assay. Through parallelism test, it was also possible to identify owl sample ideal dilution (1:16), since this dilution provided a binding percentage closer to 50% binding in the standard curve. Assay accuracy was 95.15 ± 2.9% as a mean recovery of added hormone after peak. Intra- and inter-assay variation coefficients were 2.3% and 8.2%, respectively.

**Figure 1:**
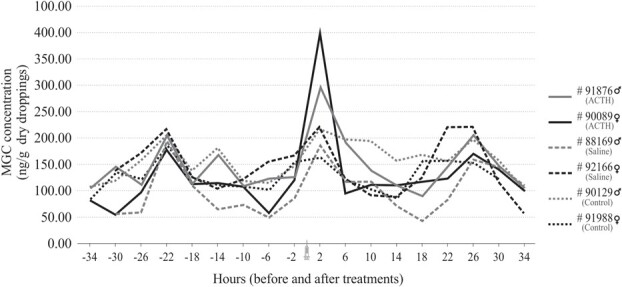
Graphic representation of glucocorticoid metabolite concentrations extracted from lyophilized fecal samples of *Megascops choliba* (MGC ng/g) during three sampling days with a collection interval of 4 hours (time −34 to 34 hours). Moment “0” is understood to be the time of administration of treatments (saline solution and/or ACTH analogue). Solid lines illustrate MGC concentrations in females and male treated with ACTH analogue at doses of 0.5 and 0.25 mg/kg, respectively. The peak values were 395.99 ng/g for the female and 295.36 ng/g for the male, which is equivalent to 3.8 and 2.2 times the basal mean value, respectively. The dotted lines correspond to the MGC concentrations of the saline-treated animals, and the dashed lines correspond to the animals in the control group.

It is important to remember that the sampling interval in which we challenged individuals comprised the period between 1700 and 2100, and that the injection occurred at 1900. Owls from the ACTH group presented a 3.8-fold increase (MGC = 395.99 ng/g) in female #90089 and of 2.2 times (MGC = 295.36 ng/g) in male #91876 compared to baseline mean levels, physiologically validating the EIA. These peaks were recorded in the first sample collected after ACTH administration (moment 2, Fig. 1). Animals in the saline and control group showed no peaks. Basal value of MGC was determined with the mean value of the concentrations contained in the 52 samples collected before treatment administration. Another 53 samples were collected after treatments, with all MGC values represented in ng/g units of each individual in Fig. 1.

### Individual and hourly variation

**Figure 2:**
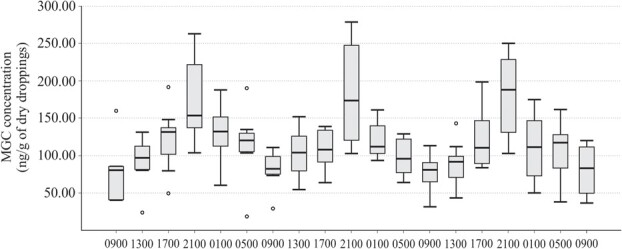
Graphic representation of glucocorticoid metabolite concentrations extracted from lyophilized fecal samples of *Megascops choliba* (MGC ng/g) during three sampling days with a collection interval of 4 hours (for three continuous days).

A total of 143 fecal samples were collected and processed to determine MGC concentrations in the nine owls evaluated in parallel with activities budget. Higher MGC concentration was exhibited at night (2100 = 173.0 ± 59.6 ng/g) compared to daytime (0900 = 80.4 ± 25.8 ng/g) (Fig. 2). Individual ID had a significant effect on MGC variation, and, therefore, it was kept as a random effect in all models (Supplementary material). The two best models were the ones with sex interacting with time and time alone, and both were not significantly different from each other based on ANOVA test. However, we opted to keep the simpler model with the lowest AIC, which is MGC ~ hour + (1|id). This model showed that the samples collected at 2100 had a greater influence on daily variation of MGC values, followed by the periods at 0100 and 0500. There was, however, a significant intercept value. In all models, there were no significant sex-related changes in MGC concentration.

### Combining activity budget and glucocorticoid metabolites

**Figure 3:**
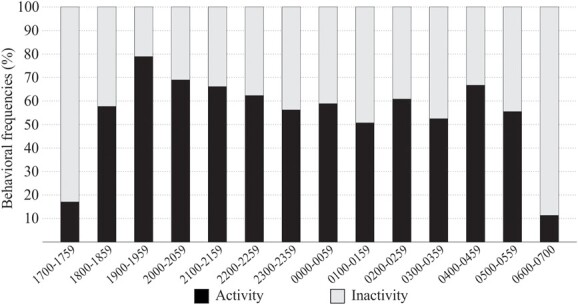
Graphic representation of the behavioral frequencies expressed by the *Megascops choliba*, during the 3 days of observation, with the evaluation period between 17:00 and 07:00. Locomotion, alert, feeding, maintenance and excretion behaviors grouped in “activity” pattern; maintenance, rest and standing behaviors grouped in “inactivity” pattern.

Each owl was evaluated for 42 hours, obtaining a total 4396 scans, or about 504 scans per individual. The behavioural frequencies recorded on three evaluation days are represented in percentage units in Fig. 3.

We found significant interaction between behavioural proportions and MGC. Specifically, rest and inactivity proportions were higher when MGC was lower. Conversely, the proportion of maintenance and activity was directly related to MGC. Considering MGC as the response and behavioural proportions as the fixed variables, we also found a significant relationship. For rest and inactivity, there was an inverse relationship, while maintenance and activity had a positive and direct relationship with MGC concentration. Addition of individual ID as the random factor did not improve model quality in any case. These combined results show that higher values of active behaviours are explained by higher MGC concentrations (Supplementary Material).

## Discussion

In this study, we validated the EIA to quantify glucocorticoid metabolites in *Megascops choliba* and verified that MGC values were influenced by animal identity (individual variation) and time of day (daily rhythm), especially at 2100, but not by sex. Also, active and inactive behaviours were found to have a significant effect on MGC concentrations.

The immunoassay evaluated proved to be reliable in the quantification of immunoreactive steroid metabolites of corticosterone, since the validation tests (e.g. parallelism, accuracy and precision) were within the limits considered acceptable in the literature (Wasser *et al.,* 2000; Möstl *et al.,* 2005; Barbosa-Moyano *et al.,* 2020). The efficacy of the immunoassay CJM006 for non-invasive monitoring of adrenal activity has been previously proven in a wide variety of taxa, including birds, reptiles, amphibians and mammals (Watson *et al.,* 2013). The EIA was also effective in differentiating MGC production according to the treatments implemented in the owls (ACTH, saline and control), confirming its physiological validity to assess the adrenal activity of *M. choliba*.

Tropical screech owls in our study showed individual variability in MGC production, similar to that described in other bird species (Ferreira *et al.,* 2015; Munteanu *et al.,* 2017; Sinhorini *et al.,* 2020). This individual difference in baseline MGC levels can be attributed to intrinsic factors, such as age, genetics and stage of life history (Boissy, 1995; Koolhaas *et al.,* 1999; Cockrem and Silverin, 2002; de Sousa *et al.,* 2015), which can result in a differentiated adrenal response when birds are exposed to stressful stimuli, as shown in different species of passerines (Stocker *et al.,* 2016; Barbosa-Moyano *et al.*, 2019). Our results also showed a daily pattern, with the highest concentrations in the evening and the lowest by morning. The daily rhythm in MGC excretion has been previously demonstrated in diurnal birds (Scheiber *et al.,* 2017; Barbosa-Moyano *et al.*, 2019); however, the patterns for nocturnal species remain unknown.

We did not detect sex-related differences in MGC, as has been seen in *Tyto alba* (Béziers *et al.,* 2020). However, in other owl species, MGC concentrations varied according to sex and reproductive stage, with higher values recorded in males at the beginning of the breeding season and higher in females during chick rearing (Wasser *et al.,* 1997; Wasser and Hunt, 2005). The lack of sex differences in MGC concentrations may be related to the fact that our study was carried during a short period in non-breeding season and may not represent a true lack of difference. In bi-parental care species (e.g. rodents Harris *et al.,* 2013; owls Béziers *et al.,* 2020), MGC is expected to be similar. *M. choliba* males have not been seen participating in egg incubation (Lima and Lima Neto, 2009; Guilherme and de Souza, 2017). Similarly, for the spectacled owl *Pulsatrix perspicillata* and the barn owl *Tyto alba*, bi-parental care is defined as females rearing the chicks, while the male brings food for them (Chaparro-Herrera *et al*., 2017; Béziers *et al.,* 2020). This pattern is also shared with other bird species, as observed in pinyon jays *Gymnorhinus cyanocephalus* (Bateman and Balda, 1973). Unfortunately, a good knowledge in the relationship between MGC and parental care of owls is still scarce in general (Lynn, 2016). Hence, it would be interesting to understand the role of glucocorticoids and parental care in this owl species.

As for behavioural analysis, individuals spent most of the daytime hours (0700 to 1800) in static perching behaviour, as has been witnessed in the wild. Wild *M. choliba* spends most of its daytime hours perched in habitats with dense and closed vegetation, as a possible strategy to minimize predation risk (Barros and Motta-junior, 2014). The highest frequency of active behaviours was described during the night period, specifically between 1900 and 2000. Detection of this time window allowed the establishment of sampling routine to capture wild *M. choliba* as, after this time, the animals hardly responded to playbacks (unpublished data). Construction of an ethogram is highly valuable as it is available for several birds, but few owl species. Exceptions include *Athene brama* (Nerlekar *et al.,* 2014), *Bubo bengalensis* (Ramanujam, 2010), *Athene blewitii* (Ishtiaq and Rahmani, 2005), *Asio flammeus* (Clark, 1975; Paulete *et al.,* 2019) and *Athene cunicularia* (Cavalli *et al.,* 2018)*.* Such scarcity may reflect the difficulty in observing nocturnal birds and the consequent lack of visibility, limiting observations to the daytime period (e.g. *Bubo scandiacus*, Boxall and Leinn, 1989). A better understanding of the behavioural repertoire of individuals facilitates assessment of the welfare of captive animals (Koene, 2013; Greggor *et al.,* 2018), allowing, for example, to identify behaviours that indicate poor environmental enrichment, such as feather plucking (Costa *et al.,* 2016; De Almeida *et al.,* 2018; Sinhorini *et al.,* 2020).

Considering MGC concentrations and activity budget, we found higher activity rates to positively correlate with high MGC levels and higher inactive behaviours with decreased MGC levels. Hence, we can assume that plasma concentrations of corticosteroids increase hours before periods of greatest activity. The timing of biochemical and physiological processes results from the interaction between the endogenous circadian rhythm and cyclic environmental factors (Cassone, 2014), giving the organism the ability to anticipate environmental variations (Schwabl *et al.,* 2016). Basal glucocorticoid concentrations in most birds can vary throughout the day, with elevations in plasma levels coinciding with the onset of active behaviours, such as locomotion and feeding (Boissin and Assenmacher, 1970; Landys *et al.,* 2006; Schwabl *et al.,* 2016). In diurnal animals, peak occurs just prior to sunrise, while in nocturnal animals, it is just prior to sunset (Touma and Palme, 2005). Interestingly, other studies, also carried out in birds, including an owl species (*Megascops kennicottii*), showed higher levels of GC during periods of inactivity (Dufty Jr and Belthoff, 1997; Belthoff and Dufty, 1998; Breuner *et al.,* 1999; Romero and Remage-Healey, 2000).

Specifically, our study revealed a direct relationship between MGC and alertness and maintenance. State of alertness is a defensive behaviour (Paulino *et al.,* 2018) and indicator of predator vigilance (Schneider and Griesser, 2013; Tätte *et al.,* 2019). Therefore, there are several examples identifying higher glucocorticoids during higher alertness or higher predation rates in several groups (mammals, Brachetta *et al.,* 2020; birds, Xu *et al.,* 2021). Such relationship relates to the predatory stress hypothesis which poses a physiological response in increased GC secretion due to predator cues (Boonstra *et al.,* 1998). Although these are captive owls, there have been moments of killing by wild opossums (Barbosa-Moyano, *pers. comm*) and such past experience can explain their alertness even in captivity. Our results showing this relationship are thus in agreement with literature findings.

The fact that higher maintenance behaviour rates correlated with increased MGC levels was an interesting finding. This category includes behaviours such as feather ruffle, shaking and preening. Preening is generally considered a comfort behaviour in many species (Henson *et al.,* 2012), owls included (e.g. Boxall and Leinn, 1989). Higher preening rates are usually present when birds are experiencing disturbances (Delius, 1988), leading to feather picking in some cases (De Almeida *et al.,* 2018). Moreover, preening behaviour correlates with increased MGC levels in some bird species, such as the goose (*Anser anser*), where geese subjected to handling stress showed increased and longer preening bouts (Kralj-Fiser *et al.,* 2010). Conversely, in other species, such as captive European starlings (*Sturnus vulgaris*), preening actually decreased after acute stressors (Nephew and Romero, 2003). However, the evaluation of behavioural and endocrine response of owls to a stressful stimulus was beyond the scope of this research.

We encourage study of new hypotheses implementing the non-invasive methods herein, which we deem highly useful to evaluate welfare of owls kept under human care. Finally, given that the relationship between activity patterns and photoperiod in basal production of glucocorticoids in birds is debatable (Romero and Remage-Healey, 2000; Pravosudov *et al.,* 2002), our study contributes to the literature body while emphasizing the knowledge gap in nocturnal bird physiology.

## Conclusion

The non-invasive hormonal assessment reported in this study confirms its applicability for the behavioural endocrinology evaluation of captive tropical screech owls. This study validated EIA for quantification of glucocorticoid metabolites; MGC values showed individual differences and also in relation to time of day, but not to sex. We also found that higher MGC concentration was present just prior to commencement of activity pattern. Moreover, increased active behaviours, particularly maintenance, were associated with higher MGC concentration. Preening behaviour, part of maintenance category, is usually regarded as a comfort behaviour, despite lack of consensus on the relationship between preening rates and MGC levels. Our results show that MGC daily rhythm is inverted in this nocturnal bird, contributing to the still immature literature on daily rhythm among nocturnal animals.

## Funding

This study was supported by grant 2021/08898-2, São Paulo Research Foundation (FAPESP) and in part by the Coordenação de Aperfeiçoamento de Pessoal de Nível Superior—Brasil (CAPES)—Finance Code 001 provided to the first author.

## Conflict of Interest

The authors have no conflicts to declare.

## Data availability

The data that support the findings of this study are available on request from the corresponding author.

## Authors Contribution

HBM collected the data; HBM and GS conceived and designed the analysis; HBM did the hormonal assays; GS performed statistical analyses; HBM and GS wrote the paper; CAO supervised the project.

## Supplementary Material

Web_Material_coad016
