# Supplementary material for: Glucocorticoid metabolites in an ex situ nocturnal bird, the tropical screech owl Megascops choliba: effects of sex, activity period and inter-individual variation
Source: Conserv Physiol. 2023 Apr 22;11(1):coad016. doi: 10.1093/conphys/coad016 (PMC10123863; doi:10.1093/conphys/coad016)
Supplement: Web_Material_coad016 [file web_material_coad016.zip › Supplementary Material 2.pdf]

Supplementary Material I: Model output testing individual identity as random effects, and time and sex as fixed effects.

**Model: Sex * Time + (1|id)**			
Predictors	Estimates	CI	p
(Intercept)	75.47	46.49 – 104.45	**<0.001**
Sex	29.77	-5.93 – 65.47	0.102
Time [21h00]	71.76	41.12 – 102.39	**<0.001**
Time [01h00]	39.53	8.90 – 70.17	**0.011**
Time [05h00]	17.35	-13.29 – 47.98	0.267
Time [09h00]	-3.06	-32.95 – 26.83	0.841
Time [17h00]	35.97	3.18 – 68.77	**0.032**
Sex * Time [21h00]	11.65	-26.45 – 49.75	0.549
Sex * Time [01h00]	-18.95	-56.67 – 18.76	0.325
Sex * Time [05h00]	-5.59	-43.30 – 32.13	0.772
Sex * Time [09h00]	-18.59	-55.12 – 17.93	0.318
Sex * Time [17h00]	-20.48	-59.97 – 19.01	0.309
**Random Effects**			
σ_2	1099.56		
$\tau_{00\ id}$	289.46		
ICC	0.21		
Marginal R^2 / Conditional R^2	0.415 / 0.537		
AIC	1637.9		
95% CI intercept	9.72 – 29.79		
95% CI residual	29.64 – 37.09		

**Model: Sex * Time**			
Predictors	Estimates	CI	p
(Intercept)	75.47	51.12 – 99.82	**<0.001**
Sex	30.58	0.47 – 60.69	**0.047**
Time [21h00]	71.76	37.32 – 106.19	**<0.001**
Time [01h00]	39.53	5.10 – 73.97	**0.024**
Time [05h00]	17.35	-17.09 – 51.78	0.323
Time [09h00]	-5.47	-39.03 – 28.09	0.750
Time [17h00]	37.12	0.31 – 73.93	**0.048**
Sex * Time [21h00]	12.46	-30.36 – 55.27	0.569
Sex * Time [01h00]	-19.76	-62.14 – 22.61	0.361
Sex * Time [05h00]	-6.4	-48.77 – 35.98	0.767
Sex * Time [09h00]	-15.83	-56.85 – 25.19	0.450
Sex * Time [17h00]	-22.44	-66.77 – 21.89	0.321
R^2 conditional / R^2 marginal	NA / 0.999		
AIC	1658		

Model: Sex + (1|id)			
Predictors	Estimates	CI	p
(Intercept)	101.44	79.56 – 123.32	**<0.001**
Sex	19.64	-7.12 – 46.40	0.150
**Random Effects**			
σ^2	2045.09		
$\tau_{00\ id}$	257.97		
ICC	0.11		
Marginal R^2 / Conditional R^2	0.036 / 0.144		
AIC	1713.4		
95% CI intercept	8.24 – 31.30		
95% CI residual	40.43 – 50.58		

**Model: Time + (1|id)**			
Predictors	Estimates	CI	p
(Intercept)	95.12	76.88 – 113.37	**<0.001**
Time [21h00]	79.02	60.55 – 97.49	**<0.001**
Time [01h00]	27.09	8.97 – 45.21	**0.003**
Time [05h00]	13.81	-4.31 – 31.93	0.135
Time [09h00]	-15.43	-32.85 – 1.98	0.082
Time [17h00]	22.02	3.53 – 40.52	**0.02**
**Random Effects**			
σ^2	1131.14		
$\tau_{00\ id}$	387.54		
ICC	0.26		
Marginal R^2 / Conditional R^2	0.364 / 0.526		
AIC	1632.5		
95% CI intercept	11.52 – 33.63		
95% CI residual	30.07 – 37.62		

Supplementary Material II: Model output testing individual identity as random effects and employing MGC as the independent variable. Significant *p* values in bold.

**Model: Proportion of Alert ~ MGC 4h prior + (1|id)**			
Predictors	Estimates	CI	*p*
(Intercept)	0.05	-0.13 – 0.24	0.559
MGC 4h prior	0.09	-0.00 – 0.18	**0.052**
**Random Effects**			
σ2	0		
$\tau_{00\ id}$	0		
Marginal R^2/Conditional R^2	0.104 / NA		
AIC with random effect	-99.4621		
AIC without random effect	-113.737		

**Model: Proportion of Feed ~ MGC 4h prior + (1|id)**			
Predictors	Estimates	CI	*p*
(Intercept)	0.02	-0.17 – 0.22	0.799
MGC 4h prior	0.01	-0.08 – 0.11	0.784
**Random Effects**			
σ2	0		
$\tau_{00\ id}$	0		
Marginal R^2/Conditional R^2	0.002 / NA		
AIC with random effect	-96.58		
AIC without random effect	-110.685		

**Model: Proportion of Rest ~ MGC 4h prior + (1|id)**			
Predictors	Estimates	CI	*p*
(Intercept)	0.72	0.32 – 1.11	**0.001**
MGC 4h prior	-0.27	-0.46 – -0.08	**0.007**
**Random Effects**			
σ2	0.01		
$\tau_{00\ id}$	0		
Marginal R^2/Conditional R^2	0.178 / 0.390		
AIC with random effect	-49.6878		
AIC without random effect	-58.0964		

**Model: Proportion of Maintenance ~ MGC 4h prior + (1|id)**			
Predictors	Estimates	CI	*p*
(Intercept)	-0.17	-0.35 – 0.02	0.073
MGC 4h prior	0.11	0.02 – 0.20	**0.015**
**Random Effects**			
σ2	0		
$\tau_{00\ id}$	0		
Marginal R^2/Conditional R^2	0.158 / NA		

AIC with random effect	-100.919
AIC without random effect	-115.28

**Model: Proportion of Stop ~ MGC 4h prior + (1|id)**			
Predictors	Estimates	CI	*p*
(Intercept)	0.26153	0.066-0.456	**0.0131**
MGC 4h prior	0.00535	-0.089-0.100	0.9128
**Random Effects**			
σ2	0		
$\tau_{00\ id}$	0		
Marginal R^2/Conditional R^2	0.000 / NA		
AIC with random effect	-94.3464		
AIC without random effect	-108.32		

**Model: Proportion of Inactivity ~ MGC 4h prior + (1|id)**			
Predictors	Estimates	CI	*p*
(Intercept)	0.46	0.27 – 0.65	**<0.001**
MGC 4h prior	-0.12	-0.21 – -0.03	**0.014**
**Random Effects**			
σ2	0		
$\tau_{00\ id}$	0		
Marginal R^2/Conditional R^2	0.159 / 0.297		
AIC with random effect	-99.813		
AIC without random effect	-112.81		

**Model: Proportion of Activity ~ MGC 4h prior + (1|id)**			
Predictors	Estimates	CI	*p*
(Intercept)	0.04	-0.06 – 0.13	0.441
MGC 4h prior	0.05	0.01 – 0.10	**0.024**
**Random Effects**			
σ2	0		
$\tau_{00\ id}$	0		
Marginal R^2/Conditional R^2	0.137 / 0.227		
AIC with random effect	-148.715		
AIC without random effect	-165.31		

**Model: Proportion of Locomotion ~ MGC 4h prior + (1|id)**			
Predictors	Estimates	CI	*p*
(Intercept)	0.19	-0.05 – 0.42	0.118
Proportion of Locomotion	0.01	-0.10 – 0.13	0.816
**Random Effects**			
σ2	0		
$\tau_{00\ id}$	0		

Marginal R^2/Conditional R^2	0.001 / 0.482
AIC with random effect	-82.2979
AIC without random effect	-85.0482

Supplementary Material III: Model output testing individual identity as random effects, and employing MGC as the dependent variable. Significant *p* values in bold.

**Model: MGC 4h prior ~ Proportion of Alert + (1|id)**			
Predictors	Estimates	CI	*p*
(Intercept)	1.78	1.49 – 2.07	**<0.001**
MGC 4h prior	1.12	-0.03 – 2.28	0.055
**Random Effects**			
σ2	0		
$\tau_{00\ id}$	0		
Marginal R^2/Conditional R^2	0.095 / 0.197		
AIC with random effect	-15.44987		
AIC without random effect	-21.44775		

**Model: MGC 4h prior ~ Proportion of Feed + (1|id)**			
Predictors	Estimates	CI	*p*
(Intercept)	2.03	1.93 – 2.13	**<0.001**
Proportion of Feed	0.37	-0.85 – 1.58	0.546
**Random Effects**			
σ2	0.3		
$\tau_{00\ id}$	0.01		
Marginal R^2/Conditional R^2	0.010 / 0.161		
AIC with random effect	-12.08181		
AIC without random effect	-17.4678		

**Model: MGC 4h prior ~ Proportion of Rest + (1|id)**			
Predictors	Estimates	CI	*p*
(Intercept)	2.17	2.06 – 2.29	**<0.001**
Proportion of Rest	-0.75	-1.26 – -0.24	**0.005**
**Random Effects**			
σ2	0.02		
$\tau_{00\ id}$	0.01		
Marginal R^2/Conditional R^2	0.177 / 0.441		
AIC with random effect	-17.26028		
AIC without random effect	-21.40605		

**Model: MGC 4h ~ Proportion of Maintenance + (1|id)**			
Predictors	Estimates	CI	*p*
(Intercept)	1.96	1.87 – 2.06	**<0.001**
Proportion of Maintenance	1.4	0.29 – 2.52	**0.015**
**Random Effects**			
σ2	0.02		
$\tau_{00\ id}$	0		
Marginal R^2/Conditional R^2	0.151 / 0.259		

AIC with random effect	-17.72103
AIC without random effect	-23.73648

**Model: MGC 4h ~ Proportion of Stop + (1|id)**			
Predictors	Estimates	CI	*p*
(Intercept)	2	1.67 – 2.33	**<0.001**
Proportion of Stop	0.19	-0.99 – 1.38	0.741
**Random Effects**			
σ2	0.03		
$\tau_{00\ id}$	0		
Marginal R^2/Conditional R^2	0.003 / 0.136		
AIC with random effect	-11.77344		
AIC without random effect	-17.39994		

**Model: MGC 4h ~ Proportion of Inactivity + (1|id)**			
Predictors	Estimates	CI	*p*
(Intercept)	2.37	2.13 – 2.62	**<0.001**
Proportion of Inactivity	-1.49	-2.57 – -0.42	**0.008**
Random Effects			
σ2	0.02		
$\tau_{00\ id}$	0.01		
Marginal R^2/Conditional R^2	0.161 / 0.390		
AIC with random effect	-18.25511		
AIC without random effect	-21.82689		

**Model: MGC 4h ~ Proportion of Activity + (1|id)**			
Predictors	Estimates	CI	*p*
(Intercept)	1.64	1.31 – 1.97	**<0.001**
MGC 4h prior	2.89	0.63 – 5.15	**0.014**
**Random Effects**			
σ2	0.02		
$\tau_{00\ id}$	0.01		
Marginal R^2/Conditional R^2	0.143 / 0.348		
AIC with random effect	-18.96084		
AIC without random effect	-21.67022		

**Model: MGC 4h ~ Proportion of Locomotion + (1|id)**			
Predictors	Estimates	CI	*p*
(Intercept)	2.06	1.84 – 2.28	**<0.001**
Proportion of Locomotion	-0.05	-1.01 – 0.91	0.914
**Random Effects**			
σ^2	0.03		

τ00 id	0
Marginal R^2/Conditional R^2	0.000 / 0.137
AIC with random effect	-11.25397
AIC without random effect	-17.52001
